# Innovative Control of Biofilms on Stainless Steel Surfaces Using Electrolyzed Water in the Dairy Industry

**DOI:** 10.3390/foods10010103

**Published:** 2021-01-06

**Authors:** Rodrigo Jiménez-Pichardo, Iriana Hernández-Martínez, Carlos Regalado-González, José Santos-Cruz, Yunny Meas-Vong, María del Carmen Wacher-Rodarte, Julián Carrillo-Reyes, Irais Sánchez-Ortega, Blanca Estela García-Almendárez

**Affiliations:** 1 Technological University of Mineral de la Reforma, Camino Providencia-La Calera 1000, Colonia Paseos de Chavarría, Mineral de Reforma, Hidalgo 42186, Mexico; rodrigo.jimenez@utmir.edu.mx; 2 DIPA, PROPAC, Faculty of Chemistry, Autonomous University of Querétaro. C.U., Cerro de las Campanas Col. Las Campanas s/n, Querétaro Qro. 76010, Mexico; irianahm@live.com.mx (I.H.-M.); regcarlos@gmail.com (C.R.-G.); jsantos@uaq.edu.mx (J.S.-C.); 3 Center for Research and Development in Electrochemistry, CIDETEQ, Parque Tecnológico Querétaro Sanfandila, Pedro Escobedo, Querétaro 76703, Mexico; yunnymeas@cideteq.mx; 4 Food and Biotechnology Department, Faculty of Chemistry, National Autonomous University of Mexico, Av. Universidad 3000, Circuito Exterior s/n, Delegación Coyoacán CDMX 04510, Mexico; wacher@unam.mx; 5 Academy Unit Juriquilla, Engineering Institute, National Autonomous University of Mexico, Blvd Juriquilla 3001, Juriquilla, Querétaro 76230, Mexico; JCarrilloR@ii.unam.mx; 6 Basic Sciences and Engineering Institute, Autonomous University of Hidalgo State, Ciudad del Conocimiento, Carr. Pachuca-Tulancingo Km 4.5, Col. Carboneras, Mineral de la Reforma Hidalgo 42184, Mexico; irais_sanchez5498@uaeh.edu.mx

**Keywords:** biofilms, electrolyzed water, stainless steel

## Abstract

Biofilms on food-contact surfaces can lead to recurrent contamination. This work aimed to study the biofilm formation process on stainless steel plates used in the dairy industry: 304 surface finish 2B and electropolished; and the effect of a cleaning and disinfection process using alkaline (AEW) and neutral (NEW) electrolyzed water. Milk fouling during heat processing can lead to type A or B deposits, which were analyzed for composition, surface energy, thickness, and roughness, while the role of raw milk microbiota on biofilm development was investigated. Bacteria, yeasts, and lactic acid bacteria were detected using EUB-338, PF2, and Str-493 probes, respectively, whereas Lis-637 probe detected *Listeria* sp. The genetic complexity and diversity of biofilms varied according to biofilm maturation day, as evaluated by 16S rRNA gene sequence, denaturing gradient gel electrophoresis, and fluorescence in situ hybridization microscopy. From analysis of the experimental designs, a cleaning stage of 50 mg/L NaOH of AEW at 30 °C for 10 min, followed by disinfection using 50 mg/L total available chlorine of NEW at 20 °C for 5 min is a sustainable alternative process to prevent biofilm formation. Fluorescence microscopy was used to visualize the effectiveness of this process.

## 1. Introduction

Milk components—mainly protein, fat, minerals, and carbohydrates—undergo structural changes during heat processing in the dairy industry. Milk fouling during heat processing can be classified as deposits type A (protein) that takes place at temperatures between 75 and 110 °C, and type B (mineral) at temperatures above 110 °C [1]. Biofouling involves the formation of biofilms on surfaces that have been conditioned with deposits. Biofilms on food-contact surfaces can lead to severe problems for the industry, such as reduced heat exchange efficiency, reduction of pipes diameter, increased internal pressure, energy required for processing, odor retention, surface corrosion, and recurrent contamination [2].

The interaction of milk biomolecules and with the surface, leading to deposits, may be affected by superficial characteristics such as chemical composition, roughness, and surface free energy, among others [3,4]. The presence of deposits may favor the adhesion of micro-organisms and possible formation of resistant structures such as biofilms. Biofilms are genetically differentiated forms of bacteria adhered to the surface through the synthesis of extracellular polymer substances (EPS), which provide increased resistance against cleaning and disinfection agents. This three-dimensional matrix protects the community against antimicrobial agents, adverse environmental conditions such as drought, and high temperatures or high pressure, and can serve as bacterial nutrient source [5,6,7].

Raw milk microbiota can be very complex and comprises lactic acid bacteria, spoilage and pathogenic bacteria, and yeasts and molds, and under appropriate conditions they may develop mixed biofilms. The analysis of mixed biofilms requires the identification and relative abundance of different species within the community. This process can be complicated, because approximately 99.9% of micro-organisms embedded in biofilms are not cultivable [8,9]. This problem may be overcome by using molecular tools such as the 16S rRNA gene sequence for bacteria, and 23S rRNA gene for yeasts. Denaturing gradient gel electrophoresis (DGGE) uses amplified polymerase chain reaction (PCR) products that may be excised from the gel and used for sequencing. Furthermore, fluorescence in situ hybridization (FISH) microscopy is an imaging technique used to study composition, growth, and development of micro-organisms in biofilms [10,11].

In the food industry, the cleaning of pipelines and production equipment can be conducted by the clean-in-place (CIP) process. It involves the sequential use of sodium hydroxide (NaOH) and nitric acid (HNO_3_) employing different washing stages, and disinfectant application; however, these chemicals are harmful to the environment. Neutral electrolyzed water (NEW) is gaining importance in the food industry mainly due to its effective antimicrobial activity, non-corrosiveness, environment-friendly properties, in situ production, and safe handling [12,13]. NEW is obtained from an electrolytic process that generates mixed species with high antimicrobial capacity, such as hypochlorous acid (HClO), hypochlorite ions (ClO^−^), chlorine dioxide (ClO_2_), and ozone (O_3_). During electrolysis, two types of water are produced, acid electrolyzed water (AcEW), and alkaline electrolyzed water (AEW). The acid fraction is produced in the anode and has a pH of 2.3–2.7, oxidation-reduction potential (ORP) >1000 mV, while AEW is produced in the cathode with pH of 10–11.5, and low ORP of −800 to −900 mV [14,15]. NEW is obtained by using a single-cell technology, exhibiting pH close to neutrality (6.0–7.0), with the advantage of avoiding equipment corrosion, and ORP of 750 mV [16,17]. The equilibrium HClO/ClO^−^ is pH-dependent, and at pH 6.5 the hypochlorous acid is the main molecule in the NEW system.

The Environmental Protection Agency (EPA, USA) allows up to 200 ppm hypochlorous acid as disinfectant of food-contact surfaces, dairy-processing equipment, and food-processing equipment [18]. It has been suggested that washing with AEW and AcEW may produce better disinfection than using hypochlorite solutions for meat, fresh produce or utensils used in food processing [19]. One report claims that the cost of CIP using electrolyzed water is 25% lower than the conventional CIP system [20]. The efficient use of NEW against free cells of *L. monocytogenes* and in biofilms has been reported [21]. This study showed that type A and type B deposits of raw milk on plates of stainless steel 304 finish 2B (SSP) and electropolished (ELP), showed different composition leading to more hydrophilic surfaces. The average roughness of any type of deposit on both stainless steel plates was higher than without deposits and allowed biofilm development. Molecular techniques allowed the study of the diversity and complexity of the biofilm population, which varied with biofilm maturation. Bacterial diversity of biofilms changed with time more predominant genera depending on the type of deposits on SSP. From experimental designs, a sustainable alternative process is proposed to prevent biofilm formation using a cleaning stage with AEW, followed by disinfection with NEW, without rinsing stages.

The aim of this work was to study the biofilm formation process on stainless steel surfaces used in the dairy industry, and the effect of a cleaning and disinfection process using alkaline and NEW.

## 2. Materials and Methods

### 2.1. Supplies

Raw milk from a local farm (Querétaro, Mexico) was used to favor the adhesion of the associated microbiota as well as biofilm formation. Commercial electrolyzed water was a gift from RusEco (Ciudad de Mexico, Mexico), AEW (Limmy) contained 340–355 mg activated NaOH/L, pH 11.9–12 and redox potential of −750 to −770 mV; NEW (Desy) contained 230–245 ppm total available chlorine (TAC), pH 6.7–7.0, and redox potential of 800–900 mV, both samples were stored under refrigeration (4 °C) in dark containers. Stainless steel plates 304 (2.5 × 2.5 cm) surface finish 2B (SSP), and electropolished (ELP) were obtained from a local supplier (Querétaro, Mexico). Bovine serum albumin, sodium chloride (NaCl), phosphoric acid, sodium hydroxide, ethylene glycol, and glycerol were purchased from Sigma-Aldrich (St. Louis, MO, USA). Plate count agar, nutrient agar (NA) and potato dextrose agar (PDA) were acquired from Bioxon (Cuatitlán, Mexico), while de Man, Rogosa and Sharpe (MRS) agar, and Oxford selective agar were from Oxoid (Basingstoke, England). Taq DNA polymerase, Tris, and agarose were purchased from Invitrogen (Carlsbad, CA, USA). The Live/Dead *Bac*Light kit was from Molecular Probes (OR, USA), whereas EUB 338, Strc-493, PF2, and Lis-637 probes were obtained from probeBase [22].

### 2.2. Stainless Steel Plates Preparation

SSP and ELP plates were washed following three steps: (1) Immersion in neutral detergent (Hyclin-plus, Hycel, Ciudad de Mexico, Mexico) at 65 °C, for 5 min, (2) Rinse with distilled water at same conditions, and (3) Rinse with distilled water at room temperature for 5 min. Then, the plates were sterilized by dry heat at 180 °C for 2 h [23], and placed in a desiccator until constant weight.

#### 2.2.1. Deposits Type A and B

All plates were submerged in 100 mL of raw milk, and heated at 70 °C for 15 min in a constant water bath (Shel-lab, VWR International, Cornelius, OR, USA), to promote protein denaturation and surface adhesion. For type A deposits the temperature increased to 90 °C, and kept for 30 min; type B deposits were obtained by increasing the temperature to 121 °C for 5 min, using an oil bath. The plates were then placed in a desiccator and the weight difference was recorded [24,25].

#### 2.2.2. Protein and Mineral Analysis of Deposits

Protein and minerals were determined on ELP and SSP with type A and B deposits [26]. Plates were washed with 10 mL of 1% (*v/v*) phosphoric acid at 50 °C for 30 min, followed by washing with 10 mL of 1% (*w/v*) NaOH at the same conditions. The wash solutions were mixed followed by total ashes determination (AOAC, 2005), and protein content using bovine serum albumin (BSA) as standard [27].

#### 2.2.3. Determination of Contact Angle and Free Surface Energy (FSE)

Contact angle was determined using three reference liquids (deionized water, ethylene glycol and glycerol), in addition to raw milk. A drop (7 μL) of each liquid was placed on the previously cleaned and degreased surface of the plate, using a drop-shaped analyzer (Mod. DSA30, Krüss, Germany), and the DSA4 software (Krüss, V1.1-02). The surface free energy of the SSP and ELP plates with and without deposits was evaluated by fitting the data to the Owens–Wendt–Rabel–Kaelble (OWRK) model [28,29]. According to these authors the FSE of a given material can be calculated using individual surface tension components of the interacting entities, according to Young′s equation (1):

(1) $$\gamma_{s}-\gamma_{SL}=\gamma_{LV}\cos\theta$$

where *γ_S_* is the surface tension, *γ_SL_* is the solid-liquid interfacial tension, *γ_LV_* is the liquid-vapor interfacial tension, and θ is the liquid contact angle. The surface tension and the FSE can be divided into dispersion or van der Waals forces and polar interactions (dipole–dipole and hydrogen bonds, among others). This interaction can be expressed as Equation (2):

(2) $$\gamma_{T}=\gamma^{D}+\gamma^{P}$$

where *γ_T_* is the total FSE, *γ^D^* is the dispersed phase surface tension and *γ^P^* is the polar phase surface tension. The OWRK model is a linear equation where the slope and the ordinate are given by the square root of the polar and dispersed phases comprising the FSE, respectively [30] (Equation (3)):

(3) $$\frac{\gamma_{LV\;}\left(1+\cos\theta \right)}{2\sqrt{\gamma_{LV}^{D}}}=\sqrt{\gamma_{s}^{P}}\sqrt{\frac{\gamma_{LV}^{P}}{\gamma_{LV}^{D}}}+\sqrt{\gamma_{s}^{D}}$$

#### 2.2.4. Roughness and Thickness of Deposits

The roughness at micrometric level of SSP or ELP plates with and without deposits was determined along a line on the surface (~2 cm) using a Profilometer (Veeco Dektak 6M, San Diego, CA, USA) and a software (Dektak, V.8.30.005). For thickness measurement the cantilever of the equipment was calibrated to make a course of around 1 cm, leaving the thickness of the deposits in the middle of this distance. Measurements were taken on the most stable area of the plot.

#### 2.2.5. Elemental Analysis and Raman Spectroscopy

Elemental analysis was conducted to complement the information obtained from the protein and mineral analyses. This analysis was performed on a sequential x-ray fluorescence spectrometer (Lab Center XRF-1800, Shimadzu, Japan). Analyzed elements were nickel (Ni), chromium (Cr) and iron (Fe) as the main components of the steel; carbon (C), oxygen (O) and nitrogen (N) as components of milk proteins, whereas phosphorus (P) and calcium (Ca) as components of milk minerals.

Raman spectroscopy may identify the type of interactions of functional groups of biomolecules on the plates surface. These interactions can be of van der Waals type, polar, and hydrogen bonds, among others that favor the attraction or repulsion of molecules or cells [31]. Spectroscopic analysis on SSP and ELP with and without type A or B deposits, was performed using a Raman spectrometer (DXR780, Thermo Fisher Scientific, Waltham, MA, USA) coupled to a 14 mV laser at 780 ± 0.2 nm. Aperture was 50 μm, and the wavelength range was 3000 cm^−1^ to 100 cm^−1^. Spectra analysis was performed using the OMNIC™ Specta software (v. 1.0.1591, Thermo Scientific, Waltham, MA, USA).

### 2.3. Analysis of Electrolyzed Water

TAC determination in NEW (pH 6.5) was carried out using the N, N- diethyl-*p*-phenylenediamine, using a handheld colorimeter (Hanna Instruments, Smithfield, RI, USA), the concentration was expressed in mg TAC/L.

The determination of NaOH (mg/L) in AEW was done by titration with 0.1 N HCl, using 1% phenolphthalein as indicator.

Oxidation-reduction potential (ORP) was determined using an Orion Star potentiometer (A211, Thermo Fisher Scientific, Marietta, OH, USA) fitted with an Ag/AgCl electrode (Orion, Thermo Scientific).

### 2.4. Biofilm Formation

For biofilm formation, sterile plates were placed in a sealed container at relative humidity >90%, under aseptic conditions in a laminar flow cabinet (Thermo Fisher Scientific, Marietta, OH, USA). Each plate was inoculated with 100 μL of raw milk, aseptically placed in the container, and incubated at 37 °C (Felisa FE-132AD, Jalisco, Mexico) for 5 d. Each plate was washed daily with 10 mL of 50 mM phosphate buffer, pH 7 to remove non-adhered cells. After rinsing, plates were inoculated with the same volume of milk to favor biofilm maturation, and returned to the same container.

Recovery of cells adhered to the surface was performed on day five. Plates were introduced into a 50 mL conical tube with 10 mL of the phosphate buffer, and mixed by vortexing (Daigger Scientific, Hamilton, NJ, USA) for 2.5 min at full speed. Serial decimal dilutions of microbial suspension in 0.85% (*w/v*) NaCl was conducted, and inoculated on different culture media using the pour plate method. The mesophilic aerobic population was determined by incubating on plate count agar at 30 °C for 24–48 h, whereas lactic acid bacteria (LAB) was determined by using MRS agar at 37 °C, for 48 h. The population of yeasts and molds was conducted using PDA at 30 °C, for 72 h, while *Listeria* sp. was enumerated using the Oxford selective agar at 37 °C during 24 h [32].

#### Cell Viability of Biofilms

SSP and ELP plates with type A or B deposits on which biofilms were developed, were analyzed for viable bacteria using the Live/Dead *Bac*light bacterial viability kit. This kit comprises a mixture of two fluorochromes, SYTO 9 (6 µM) and propidium iodide (PI, 30 µM), both fluorochromes were mixed 1:1 (*v/v*) to reach 100 µL, and used to cover the plates on the fifth day of biofilms formation, and allowed to stand for 15 min in the dark. When exposed to excitation wavelength of 480/500 nm, emission showing fluorescent green color is attributed to intact cell membranes, whereas fluorescent red color is associated with damaged cell membranes [33]. Plates without biofilms were used as control, followed by observation under a fluorescence microscope (Zeiss, Axioskop 40, FICT filter, Göttingen, Germany), fitted with a camera (Axio CamMRc, Zeiss), and Zeiss ZEN pro 2012 digital imaging software (v. 1.1.2.0.).

### 2.5. Molecular Studies of Biofilms

#### 2.5.1. Fluorescent in Situ Hybridization (FISH) Microscopy

Once biofilms were established on the plates, they were washed twice with 50 mM phosphate buffer, pH 7.2, and fixed by immersing the plates in 3.7% formaldehyde solution at 4 °C, for 12 h. Cells were dehydrated with successive immersions in different concentrations of ethanol (50, 80 and 95% *v/v*) for 3 min each.

The probes used were EUB 338 [34], Strc-493 [35], PF2 [36], and Lis-637 [37] (Table 1). A 1:8 (*v/v*) dilution of each probe was prepared separately in hybridization buffer (0.9 M NaCl, 0.1 M Tris [pH 7.2], 0.1% (*w/v*) sodium dodecyl sulfate (SDS), and incubated for 2 h at 46 °C. After hybridization, the plates were washed with Oether solution (4 M NaCl, 0.1 M Tris [pH 8.0], 0.5 M EDTA, 10% [*w/v*] SDS) at 46 °C; the plates were blot-dried and placed in the fluorescence microscope (Zeiss), FICT Filter, at wavelength of 530 nm [38].

#### 2.5.2. DNA Extraction and Denaturing Gradient Gel Electrophoresis (DGGE)

In this study, we used SSP plates only, with dimensions of 10 cm x 10 cm exhibiting type A or B deposits, in addition to biofilms formed at 1, 3, and 5 days. The DNA was extracted from each plate using the power soil DNA isolation kit (Mo Bio Laboratories, Carlbad, CA, USA) following the manufacturer’s protocol, with modifications. The DNA quality and concentration were measured by the nanoDrop 1000 spectrophotometer (Thermo Fisher Scientific, Marietta, OH, USA), while integrity was observed using 1% (*w*/*v*) agarose gel.

The bacterial 16S rDNA amplification was carried out by a nested PCR using Taq DNA polymerase. Primers used in the first round were 27F (5′-GTTGATCCTGGCTCAG-3′) and 1492R (5′- ACGGYTACCTTGTTACGACTT-3′), the reaction conditions were: 94 °C for 3 min; 35 cycles of 94 °C for 60 s, 45°C for 60 s, 72 °C for 1 min, and finally 72 °C for 10 min. The second-round primers were 357F-GC (5′GC-clamp-CCTACGGGAGGCAGCAG-3′) and 907R (5′-CCGTCAATTCMTTTGAGTTT-3′), the touchdown PCR conditions were 96 °C for 4 min; 10 cycles of 94 °C for 30 s, 61 °C for 1 min, decreasing 1 °C for each annealing cycle to 56 °C, then 72 °C for 1 min; plus 20 cycles at constant annealing temperature of 56 °C, and finally 72 °C for 7 min [39]. To obtain enough amplicons concentration all DNA was mixed and purified using the Wizard^®^ SV gel and PCR clean-up kit (Promega, Madison, WI, USA), eluting the purified DNA in 35 μL of nuclease-free water.

DGGE was performed with DCode Universal Mutation Detection System (Bio-Rad, Hercules, CA, USA). The PCR products were separated in 8% polyacrylamide gels (1 mm thick) in 0.5X TAE buffer with a linear denaturing gradient (urea-formamide) from 30% to 60% (*w*/*v*). Electrophoresis was carried out at 60 °C, 70 V, during 16 h. DGGE bands were developed with AgNO_3_ [39]. The gel bands were digitalized (GelDoc Imaging system, Bio-Rad) and analyzed with the Quantity One Software (Bio-Rad), generating a matrix with the intensity for each detected band. The Shannon–Wiener (*H′*) and Equitability (J′) indexes were calculated to obtain the relative intensity of each band [40] (Equations (4) and (5)):

(4) $$H^{\prime }=\;-\;\overset{i=n}{\underset{i=1}{\sum }}pi\;\ln\;pi$$

where *pi* is the proportion of intensity of band *i* relative to the total intensity of the bands. This index spans from 1 when all species are equally represented, to 0 when there is a dominant species.

(5) $$J^{\prime }=\;\frac{H^{\prime }}{H_{max}^{\prime }}$$

where *H′_max_* = ln S; S = number of gel bands

The analysis was computed with the software R, using the “vegan” and “cluster” packages. Each band was excised from the gel and the DNA was eluted in 35 µL of deionized water, then a freeze-thaw process was applied (−20 °C for 2 h, then heating at 60 °C for 30 min, three times) before storage at 4 °C. The eluted DNA was reamplified by PCR using primers 357F without GC-clamp, and 907R. The PCR products were sent for sequencing to RTL Genomics (Lubbock, TX, USA).

### 2.6. Cleaning and Disinfection Process

The complete cleaning and disinfection process consisted of three steps; rinse with phosphate buffer (pH 7) after 5 days of biofilm maturation, then cleaning with AEW, and finally disinfection with NEW, under static conditions to simulate low flow zones in the equipment.

A complete 2^3^ factorial design with four center points (Table S1) was used to determine the best conditions for the cleaning stage (using AEW). However, the microbial population was similar for all AEW concentrations tested (Table S2), thus further evaluation was performed employing an unifactorial design using AEW at concentrations <300 mg/L NaOH, keeping constant the final disinfection process of 200 ppm of NEW, at 20 °C for 5 min. Additionally, once the adequate cleaning step was found another unifactorial design was used, to determine if lower concentrations of NEW were adequate for disinfection. For all treatments, the response variable was the microbial population by plate count agar for 24 h at 30 °C.

### 2.7. Statistical Analysis

All experiments were conducted in triplicate and all data were expressed as the mean ± standard deviation. Experimental designs were analyzed using the JMP software v.8 (SAS Institute, Charlotte, NC, USA). Fitting parameters were compared by analysis of variance (ANOVA), and significant difference between treatments was performed by the Tukey test with *p* < 0.05.

## 3. Results and Discussion

### 3.1. Composition of Deposits

Type A deposits were mostly comprised by proteins (50–70%), whereas minerals were mostly found in type B deposits (72.6 ± 3.3%, *w*/*w*). Similar composition was observed for ELP and SSP, being proteins the main component of type A deposits, and minerals for type B deposits (Table 2). Among milk proteins, β-lactoglobulin is the most important for deposit formation because it is highly thermolabile, and when denatured it can interact with other milk components forming aggregates that later precipitate, and form deposits or interact directly with the surface. Subsequent layers are formed favored by an increased number of possible aggregated protein and minerals interactions [1].

### 3.2. Elemental Analysis of Deposits

Elemental analysis showed that there was no significant difference on the composition of SSP and ELP with and without deposits, with about 70 ± 1% (*w*/*w*) iron, 18 ± 1% (*w*/*w*) chromium, 8 ± 0.5% (*w*/*w*) nickel, and 0.05±0.01% phosphorus in agreement with ASTM A276-06 standard [41]

Elements such as manganese, sulfur, and silicon were not detected by the equipment used, because of very low penetration. Furthermore, the presence of a layer of chromium oxide on the surface makes X-rays penetration difficult, and this explains why carbon was not detected.

**Figure 1:**
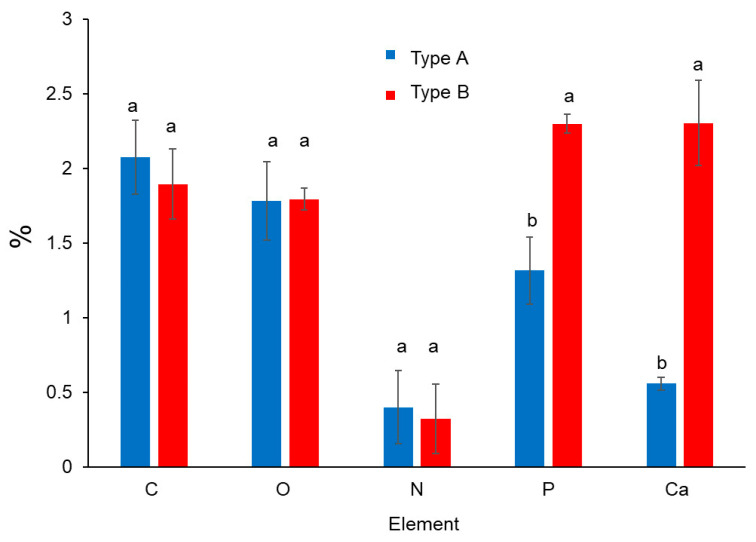
Organic components of proteins (C, O, N) and minerals (P, Ca) of deposits type A and B on SSP plates.

The elements of proteins and minerals were only analyzed in deposits on SSP surface. The main elements comprising proteins, i.e., carbon, oxygen and nitrogen did not show significant difference (*p* < 0.05) between type A and B deposits, whereas phosphorus and calcium concentration revealed significantly higher content (*p* < 0.05) of type B deposits (Figure 1), because of more mineral composition (Table 2).

### 3.3. Surface Characterization of Plates with and without Deposits

#### 3.3.1. Contact Angle and FSE

Stainless steel can be considered a partially hydrophobic surface, since the contact angles are relatively high (>50°) for all tested liquids (Table 3). These results agree with [1] (p. 30), who mentioned that the presence of the passivated layer on this surface reduces the possibility of polar or electrostatic interactions that favor molecular attraction. Plates with type A deposits show relatively lower contact angle than type B deposits, because the former are mostly comprised by proteins, which have greater number of side chain groups capable of interaction with other molecules.

The contact angle between a liquid and a surface allows determination of whether the liquid can wet the surface with which it is in contact. The surface will be hydrophilic when contact angle is <90° representing a greater interaction of the liquid with the surface. Conversely, if this value is >90° there is less interaction between the liquid and the surface, resulting in a hydrophobic surface.

In summary, the most hydrophilic surfaces were those presenting type A deposits, followed by those with type B deposits, and finally the plates without deposits, for both stainless steel types (Table 3).

There are different methods to determine FSE, and it is important not to use a single method so as to have enough information to obtain more reliable conclusions. In this case, the state equation was used, which only considers the contact angle of a liquid of known surface tension. On the other hand, the OWRK method uses contact angles obtained using polar and non-polar liquids to separate the dispersed and polar phases from the FSE, where the polar part determines the hydrophilicity of the surface, and mostly influences the wettability [30]. Results using the OWRK method agreed with those determined from the state equation.

As expected, significantly higher free energy (*p* < 0.05) of plates with type A or B deposits was observed than that of plates without deposits (Table 4). These results were attributed to the presence of biomolecules and minerals on the plates surface, which increased the hydrophilicity due to higher number of reactive groups interacting with other molecules [4]. According to the state equation, it can be considered that plates with attached deposits are significantly (*p* < 0.05) more hydrophilic than plates without deposits, and this may promote microbial adhesion.

The difference between free energy values of SSP and ELP may be due to the composition of the deposits formed on the plates, as shown in Table 2. [42] (p. 246), mentioned that the polar component of the free energy contributes to increased wettability, indicating that type A or B deposits of SSP have larger number of organic groups for possible interactions. However, the polar fraction of ELP decreased, especially for type B deposits, which may be explained by the high mineral and low protein composition (Table 2).

The dispersed fraction showed significantly larger values (*p* < 0.05) for both deposits on SSP and ELP, which refers to the groups capable of forming weak van der Waals interactions. Thus, an increase in this fraction indicates more induced dipole-induced dipole, dipole–dipole, and induced dipole–dipole interactions [43].

#### 3.3.2. Surface Topography and AFM

Roughness can be described by parameters such as Ra (arithmetic mean height), Rms (quadratic mean roughness), and Rt (difference between the highest peak and the deepest valley, through the exploration area), among others. Average roughness (Ra) of SSP with type A or B deposits was significantly higher (*p* < 0.05) than that of the plates without deposits (30.7 ± 0.06 μm). Similarly, ELP roughness for both type of deposits was higher than that of plates without deposits (0.11 ± 0.01 nm) (Table 5). As expected, the Ra value of ELP was 279 times lower than that of SSP. On the other hand, it is clear that the thickness of any type of deposits on the highly smooth ELP was much thinner than those of the SSP (Table 5). Surface roughness represents an increase in available surface area and may promote macromolecules and microbial adhesion, as well as providing shear protection [44]. One study has reported a positive correlation between adhesion and surface roughness [45], while other reports claim that there is no such correlation [46,47].

**Figure 2:**
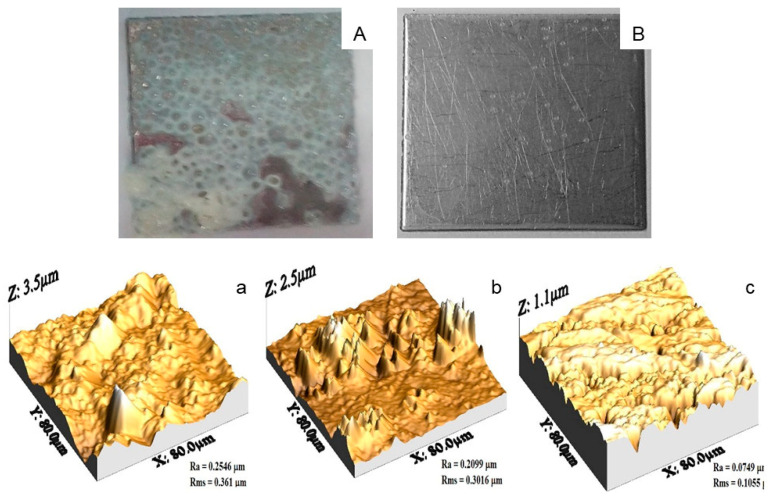
Appearance of SSP with deposits type (**A**) (panel A), and type (**B**) (panel B). AFM micrographs of SSP plates: (**a**) type A deposits; (**b**) type B deposits; (**c**) SSP without deposits.

The appearance of type A deposits on SSP was white, soft, and spongy due to the aggregation of denatured proteins and gel formation, to which milk minerals and other components may diffuse (Figure 2A), and agrees with other reports [31,48]. SSP with type B deposits show low solids concentration, which according to Table 2 was attributed to the predominant mineral composition, Figure 2B. The topography of any surface can be represented by series of peaks and valleys varying in profile and uniformity that are visualized for SSP with type A and B deposits in Figure 2a,b, whereas Figure 2c shows the SSP without deposits.

#### 3.3.3. Raman Spectroscopy of Deposits

**Figure 3:**
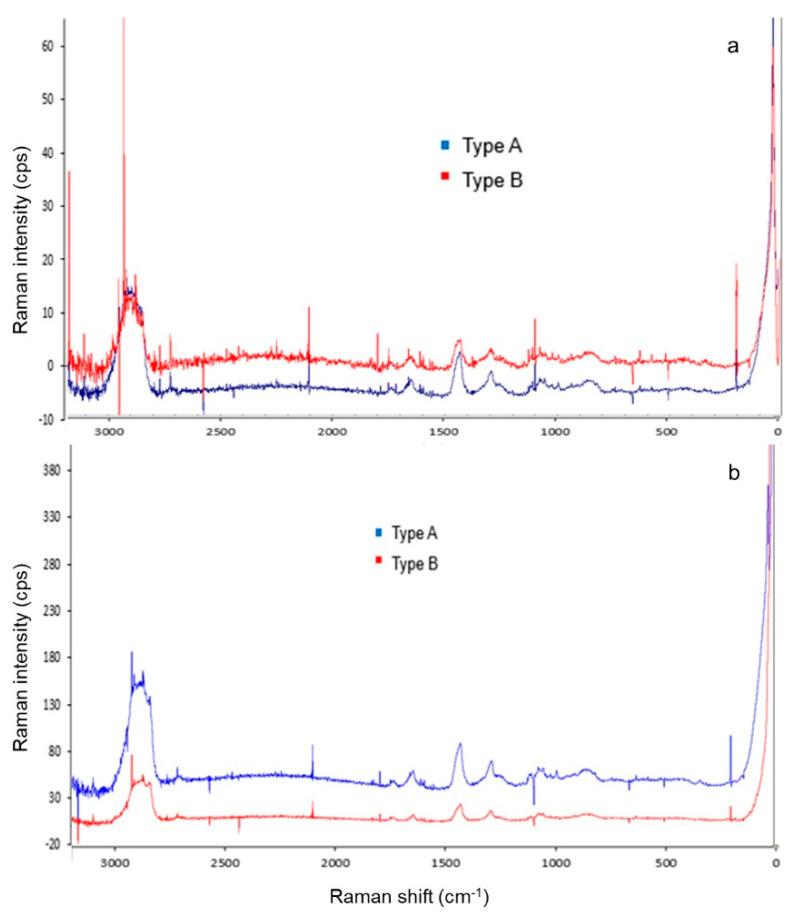
Raman spectra obtained from the analysis of the SSP (**a**), and ELP (**b**) with deposits type A (blue line) or type B (red line).

The analysis of the Raman spectra of milk deposits on the plates surface was performed by comparing the peaks reported by [49]. Peaks below 3000 cm^−1^ match those shown by proteins, fatty acids, and lactose. Peaks at 2900 cm^−1^ and 1450 cm^−1^ correspond to casein, while the peak at 1350 cm^−1^ corresponds to fatty acids observed in both type of deposits of SSP and ELP (Figure 3a,b).

The narrow signals at 1100 cm^−1^ correspond to the C-C, C-O, and C-CH_3_ bonds of lactose, which was only observed in both types of deposits of SSP (Figure 3a).

Visible signals in the 1000 cm^−1^, 560 cm^−1^, and 420 cm^−1^ correspond to calcium phosphate [50] (p. 917), and [51] (p. 12226); but only the signal at 560 cm^−1^ was observed for type B deposit on SSP, which was attributed to the high mineral concentration of this deposit (Figure 3a). The calcium signal (200 cm^−1^) was higher for type B deposit on SSP than either deposit on ELP (Figure 3a,b).

Raman spectra obtained for type A (red line) and type B (blue line) deposits on ELP are shown in Figure 3b. The spectra of both type of deposits on SSP and ELP were similar. However, both types of deposits on ELP showed lower noise that produced more defined peaks, because of lower concentration of organic matter leading to low interference [52].

### 3.4. Biofilms

#### 3.4.1. Biofilms Formation on SSP and ELP

**Figure 4:**
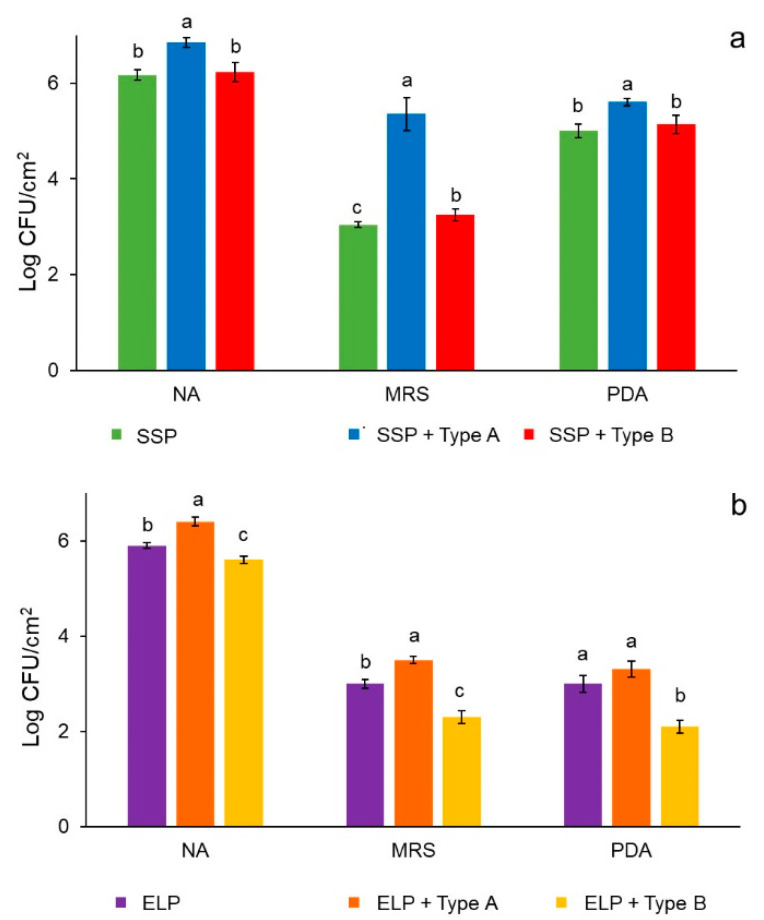
Microbial population recovered from 5-day biofilms formed on the surface of SSP plain and with type A or B deposits (**a**), and ELP plain and with type A or B deposits (**b**). NA = nutrient agar, MRS = de Man Rogosa Sharpe agar, PDA = potato dextrose agar.

Biofilms from milk microbiota were developed on type A and B deposits of SSP and ELP. The microbial population recovered from the plates showing type A deposit was higher for any of the three culture media tested (NA, MRS, and PDA) than plates with type B deposits or without deposits (Figure 4a,b). LAB (grown in MRS) and yeasts (grown in PDA) reached a larger population in SSP than ELP. This effect can be associated with the surface characteristics of the steel plates, ELP has a smother surface, but after deposits formation the roughness increased more than 30 times for any type of deposit, and became more hydrophilic, which may have favored microbial adhesion.

#### 3.4.2. Viability of Cells within the Biofilms

**Figure 5:**
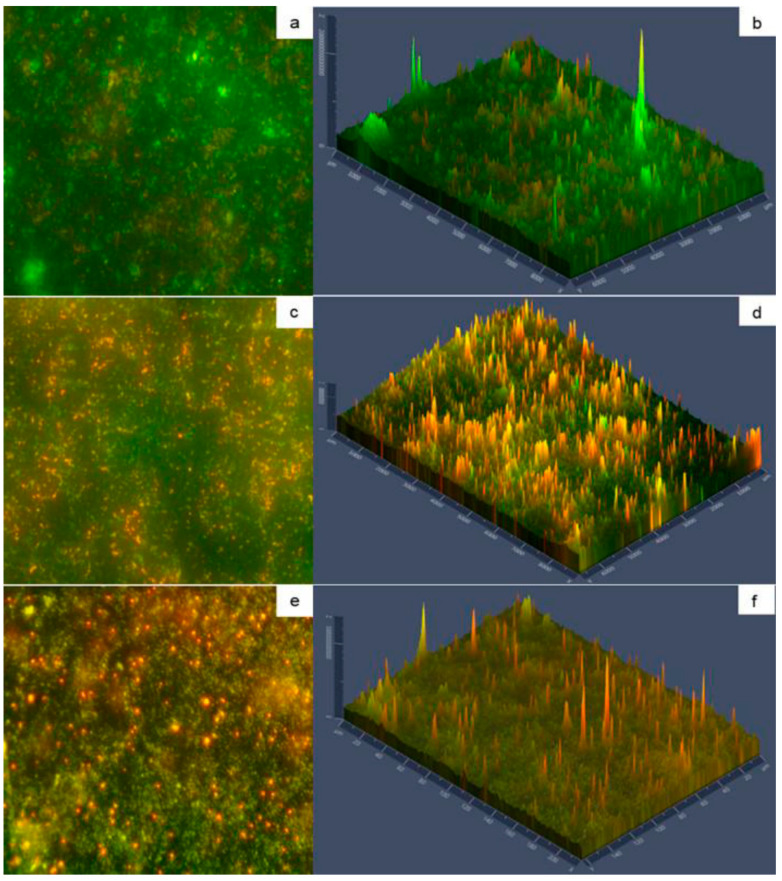
Micrographs of SSP with biofilms on type A deposits (**a**,**b**) and type B deposits (**c**,**d**); ELP with biofilms on type A deposits (**e**,**f**). Green fluorescence indicates viable cells, while red fluorescence indicates damaged cells. Panels (**a**,**c**,**e**) show 40X magnification; Panels (**b**,**d**,**f**) show fluorescence intensity.

The transition of reversible to irreversible attachment of micro-organisms in biofilms is related to the synthesis of extracellular polymeric substances that not only help in the attachment process but also protect the cells from environmental fluctuations [53,54] This leads to the formation of microcolonies, which coalesce to form a mature biofilm. The micrographs of biofilms on SSP with type A deposits show more green fluorescence (Figure 5a,b) than type B deposits (Figure 5c,d), indicating higher number of viable cells in the mature biofilm [55]. This can be associated with more surface area and higher protein concentration that can support microbial growth for longer time. In relation to ELP with type A deposits viable cells were visualized as small clusters of scattered damaged cells across the plate surface, suggesting viability loss due to the surrounding environment with depleted nutrients (Figure 5e,f).

The morphology of biofilm matrix can be visualized by the fluorescence intensity shown in Figure 5 (panel b, panel d, and panel f) indicating that more damaged cells are in the external biofilm layer. Bacteria may leave the biofilm using at least three ways, desorption, detachment, and dispersion. The former two mechanisms are passive cells release, whereas dispersion is an active phenotypic switch, allowing them to leave the biofilm. The cells sense signals that are transduced through regulatory networks that facilitate cellular release, which can be native or environmental [56]. The micro-organisms from mature biofilms can be detached due to disruptive factors among others, leading to recurrent contamination of the food-processing environment [6].

#### 3.4.3. Fluorescent Hybridization in Situ (FISH) Microscopy

**Figure 6:**
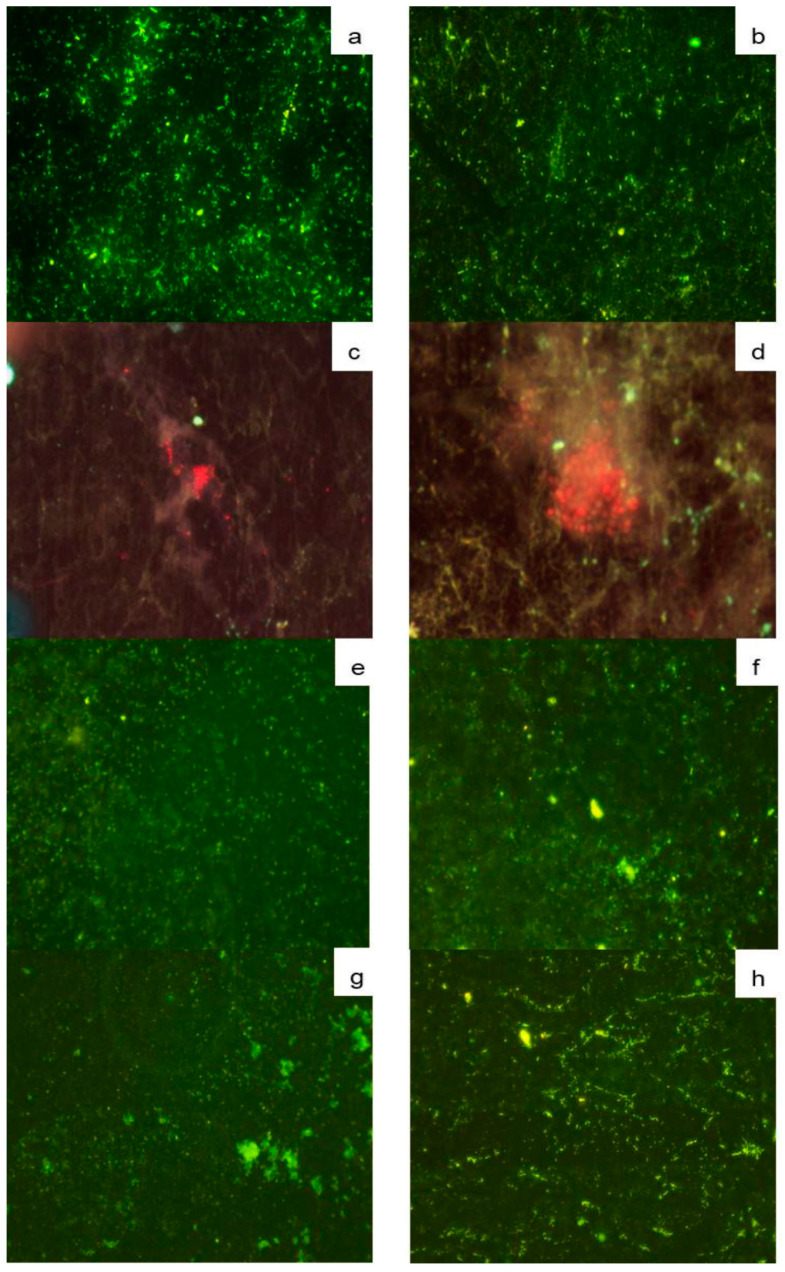
FISH micrographs (40X) of SSP plates with biofilms on type A (panels **a**,**d**,**e**,**g**) and B deposits (panels **b**,**d**,**f**,**h**), hybridized with the probes EUB338 (**a**,**b**); PF2 (**c**,**d**); Strc493 (**e**,**f**) and Lis-637 (**g**,**h**).

This microscopy technique allows an understanding of the complexity of micro-organisms present in the biofilms. Bacteria were identified with the EUB338 probe and showed cocci and bacilli morphologies on SSP plates with type A deposits (Figure 6a), whereas scattered cells were observed in type B deposits (Figure 6b), which agrees with the bacterial population reported in Figure 4a.

The elongated and ovoid characteristic shape of yeast cells on SSP was observed as red fluorescence using the PF2 probe, surrounded by a diffuse extracellular material for type A (Figure 6c), and type B deposits (Figure 6d). LAB are one of the most representative groups present in milk microbiota, which hybridized for both types of deposits with probe Strc493 specific for *Streptococcus* spp. and *Lactococcus* spp., showing green fluorescence (Figure 6e,f). *Listeria* spp. in both types of deposits on SSP plates, represent a potential health hazard of biofilms in the dairy industry, which was identified by the probe Lis-637 (Figure 6g,h).

This finding highlights the importance of performing an adequate cleaning and disinfection process of equipment and utensils used in the dairy industry, to prevent foodborne diseases that may also represent large economic losses [57,58]. Among listerial strains, *L. monocytogenes* has been reported as highly competitive against common milk microflora [59]. Therefore, these results show the complexity of biofilms arising from milk microbiota.

#### 3.4.4. Denaturing Gradient Gel Electrophoresis (DGGE)

**Figure 7:**
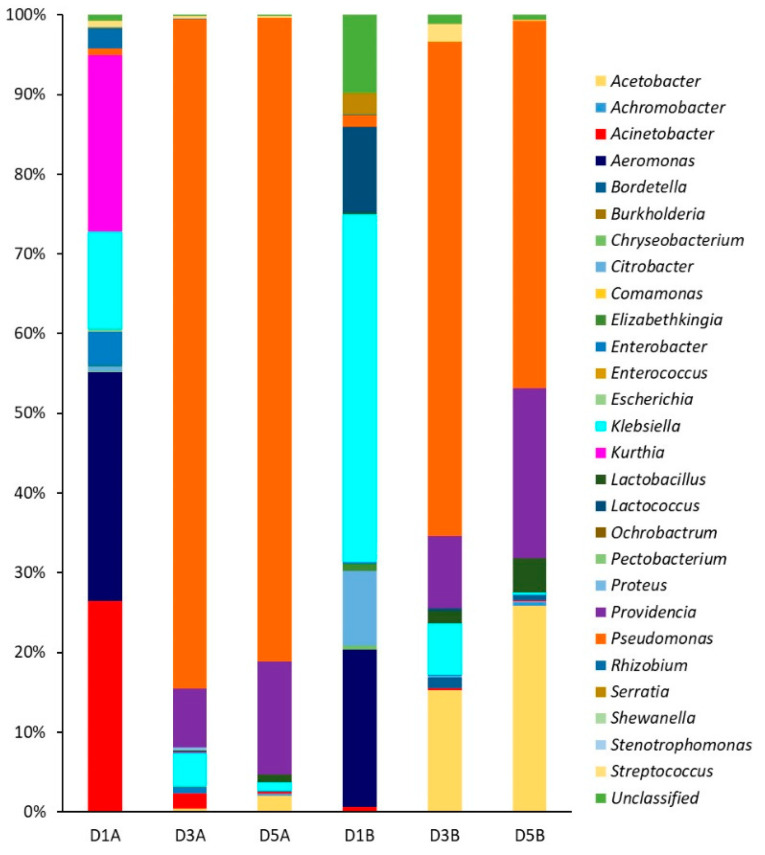
Relative abundance of species on DGGE gel at different biofilm maturation time and type of deposit on SSP. Deposits type A at day 1 (D1A), day 3 (D3A), day 5 (D5A). Deposits type B at day 1 (D1B), day 3 (D3B), day 5 (D5B).

The genetic biodiversity was determined by DGGE analysis of the 16S ribosomal DNA PCR products using specific primers for the bacterial domain. DGGE is a molecular technique used to study the microbial biodiversity of biofilms; bands from the gel can be excised and sequenced to identify community members. After purification, the DNA concentration was around 60 ng/μL, while the A_260_/A_280_ values ranged between 1.70 and 2.0 for days 1–5 of type A and B deposits. The PCR products were subjected to DGGE and all bands were excised (results not shown). Sequence analysis revealed a diverse consortium comprising up to 27 genera that changed according to biofilm maturation day on SSP (Figure 7). The relative abundance for each microbial group was calculated to obtain the Shannon–Wiener index (H’) and Equitability index (J′) (Table 6). Values of H’ between 1 and 3 indicate low diversity, while values >3 indicate high diversity; additionally, J′ = 0 indicates dominance of one or more species, whereas J′ = 1 indicates that all species are equally represented.

The bacterial diversity of biofilms on type A deposits decreased from days 1 to 5 according to the Shannon–Wiener index, and confirmed by reduction of the Equitability index. Biofilms on days 3 and 5 showed predominance of *Pseudomonas* genus (Figure 7), and a reduction of diversity as indicated by the J′ values (Table 6). *Pseudomonas* are commonly found in dairy plants and many isolates can form biofilms [60] as shown here. In particular, biofilms of *Pseudomonas aeruginosa* of dairy origin have shown multiple resistance to chemical treatment [61]. On the other hand, biofilms on type B deposits showed less biodiversity changes with maturation time. On days 3 and 5 three genera were dominant, being the most abundant *Pseudomonas*, followed by *Streptococcus* and *Providencia*, while equitability remained constant (Table 6, Figure 7). This finding confirms that biofilms comprise mixed bacterial populations, while the most predominant groups are the best adapted to the environmental conditions. There are reports showing the importance of biofilm development during storage and handling of raw milk, the genera involved, and their influence on the quality and safety of final products [62,63].

### 3.5. Cleaning and Disinfection Process

Similar population reduction in all treatments of the factorial experimental design were obtained (Table S2). However, concentration of AEW was the factor with greater influence on the microbial population, showing *p* = 0.1086 (Table S3). Given the similar population reduction smaller AEW levels were used for cleaning, applying an unifactorial design (50, 100 and 200 mg/L NaOH) (Table 7). Time and temperature were kept constant at the lowest level (10 min and 30 °C) (Table S1), and the disinfection stage at 200 ppm of NEW. The initial microbial population recovered from the mature biofilm was 6.97 ± 0.03 log CFU/cm^2^, and sterile distilled water was used as control. The population reduction was significantly different from the control (*p* < 0.05), with R^2^ = 0.99, but the Tukey test indicated no significant difference (*p* > 0.05) between treatments (Table 7).

To test the effect of lower NEW concentrations in the disinfection stage another unifactorial design was conducted, fixing the cleaning stage at the lowest AEW concentration of 50 mg/L NaOH (Table 8), while the TAC concentration of NEW ranged from 50 to 200 mg/L (Table 8). Time and temperature were as previously defined (Section 2.6), and the initial microbial population recovered from the mature biofilm was 5.67 ± 0.11 log CFU/cm^2^, using distilled water as control.

Electrochemically generated NaOH from AEW has more symmetric and weak hydrogen bonds [64], and shows a surface-active effect because of water molecules dissociation forming unstable complexes that are in a metastable state making them highly reactive [65].

**Figure 8:**
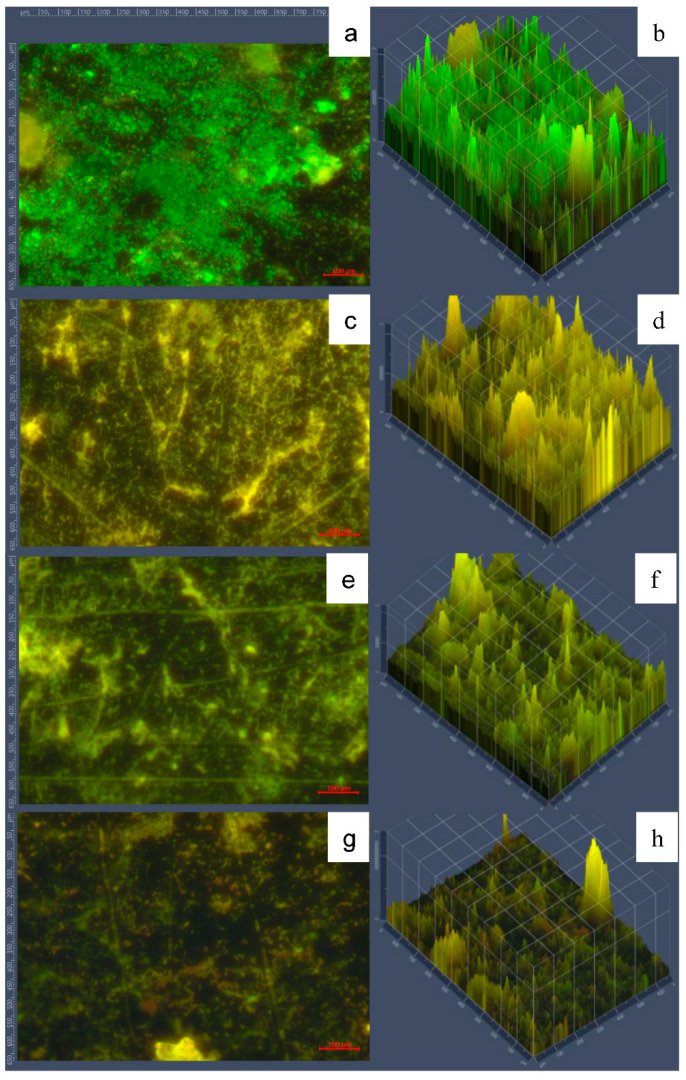
Micrographs of SSP with biofilms on type A deposits. Panels (**a**,**b**) show 5-day biofilms. Panels (**c**,**d**) show the cleaning stage of 50 mg NaOH/L of AEW at 30 °C for 10 min, only. Panels (**e**), f show disinfection using 50 mg/L total available chlorine of NEW at 20 °C for 5 min, only. Panel (**g**,**h**) show cleaning followed by disinfection stages. Green fluorescence indicates viable cells, while red fluorescence indicates damaged cells. Panels (**a**,**c**,**e**,**g**) show 40X magnification; panels (**b**,**d**,**f**,**h**) show fluorescence intensity.

According to Tukey’s test all treatments were similar (*p* > 0.05), showing a microbial population reduction close to 5 log (Table 8), after the sequential cleaning and disinfection stages. Therefore, from these results a cleaning stage is proposed of 50 mg NaOH/L of AEW at 30 °C for 10 min, followed by disinfection using 50 mg/L TAC of NEW at 20 °C for 5 min, without any rinsing stage. The effectiveness of this process was visualized using fluorescence microscopy (Figure 8).

The mature biofilms (5 days) showed high viable population observed mostly as green fluorescence (Figure 8a,b). None of the individual cleaning or disinfection stages alone were as efficient as the combined sequential stages (Figure 8g,h), where less fluorescence and extensive destruction of 3-dimensional cellular structure can be visualized.

## 4. Conclusions

Type A and B deposits of raw milk under static conditions on SSP and ELP showed different composition, leading to more hydrophilic surfaces. The average roughness of any type of deposit on both stainless steel plates was higher than without deposits, and allowed biofilm development. Molecular techniques allowed the study of the diversity and complexity of the biofilm population, which varied with biofilm maturation. *Listeria* sp. was found in both types of deposits on SSP plates. Bacterial diversity of biofilms changed with time to more predominant genera depending on the type of deposits on SSP. From experimental designs, a sustainable alternative process is proposed to prevent biofilm formation using a cleaning stage of 50 mg/L NaOH of AEW at 30 °C for 10 min, followed by disinfection with 50 mg/L TAC of NEW at 20 °C for 5 min, without any rinsing stage.

## Figures and Tables

**Table 1:** Characteristics of the probes used for FISH microscopy.

Probe	EUB338	PF2	Strc-493	Lis-637
Specificity	90% of bacteria	All yeast	*Streptococcus* spp. *Lactococcus* spp.	*Listeria* spp, except *L*. grayi
Target Gen	16S rRNA	18S rRNA	16S rRNA	16S rRNA
Position	338–355	618–636	493–511	637–657
Sequence	5′- GCT GCC TCC CGT AGG AGT -3′	5′- CTC TGG CTT CAC CCT ATT C -3′	5′- GTT AGC CGT CCC TTT CTG G -3′	5′- CAC TCC AGT CTT CCA GTT TCC-3′
Fluorophore	Fluorescein	Texas red	Cy3	Fluorescein

**Table 2:** Composition of Type A and B deposits on stainless steel plates surface finish 2B (SSP), and electropolished (ELP).

Deposits	Protein (% *w/w*)	Minerals (% *w/w*)
Type A (SSP)	48.0 ± 3.5 a	44.0 ± 6.6 b
Type B (SSP)	27.0 ± 3.6 b	72.0 ± 3.3 a
Type A (ELP)	55.0 ± 4.7 a	35.0 ± 3.0 b
Type B (ELP)	28.0 ± 1.2 b	70.0 ± 1.2 a

Different letters in the same column indicate significant difference (*p* < 0.05).

**Table 3:** Contact angle for different model liquids on SSP and ELP with and without deposits.

Surface	Water	Ethylene Glycol	Glycerol	Raw Milk
SSP	52.7 ± 1.1 a	59.9 ± 0.6 a	66.5 ± 0.6 b	48.2 ± 0.9 a
Type A	24.2 ± 0.9 b	40.6 ± 1.2 b	66.2 ± 1.8 b	23.4 ± 1.7 b
Type B	23.1 ± 1.2 b	46.2 ± 0.3 b	74.2 ± 1.6 a	26.1 ± 0.9 b
ELP	76.2 ± 0.9 a	51.0± 0.4 a	76.6± 0.4 a	67.3 ± 0.5 a
Type A	25.5± 1.8 c	42.1± 1.1 b	72.2 ± 0.8 b	22.6 ± 1.2 c
Type B	38.2 ± 1.2 b	47.6± 0.8 b	77.7 ± 0.9 a	41.9 ± 1.4 b

Columns with different letters (a–c), indicate significant difference relative to plates without deposits (*p* < 0.05).

**Table 4:** Surface free energy (mN/m) of SSP and ELP plates, with and without type A or B deposits.

Surface	State Equation	Total OWRK *	Polar Fraction	Dispersed Fraction
SSP	49.1 ± 3.6 b	55.7 ± 1.6 b	1.0 ± 0.1 c	54.7 ± 1.5 b
Type A	62.2 ± 5.9 a	122.5 ± 12.5 a	4.6 ± 0.6 b	117.9 ± 11.9 a
Type B	61.9 ± 0.3 a	125.5 ± 12.9 a	12.4 ± 0.4 a	113.1 ± 12.0 a
ELP	36.7 ± 0.9 c	46.4 ± 4.4 c	33.7 ± 1.9 a	12.7 ± 2.5 c
Type A	62.1 ± 1.5 a	153.2 ± 5.2 a	18.8 ± 3.4 b	134.4 ± 1.8 a
Type B	54.7 ± 2.3 b	108.9 ± 3.6 b	1.9 ± 0.1 c	107.0 ± 3.5 b

* OWRK: Owens–Wendt–Rabel–Kaelble. Columns with different letters (a–c) indicate significant difference relative to plates without deposits (*p* < 0.05).

**Table 5:** Surface roughness of SSP and ELP plates with and without type A and B deposits.

Surface	Ra (nm)	Rms (nm)	Thickness (nm)
SSP	30.70 ± 0.06 c	41.30 ± 0.01 c	ND
Type A	63.10 ± 0.08 a	84.60 ± 0.02 a	100.50 ± 0.01 a
Type B	42.20 ± 0.20 b	56.10 ± 0.6 b	80.50 ± 0.03 b
ELP	0.11 ± 0.01 c	0.15 ± 0.02 c	ND
Type A	13.15 ± 0.15 a	16.83 ± 0.13 a	14.83 ± 2.17 a
Type B	4.03 ± 0.13 b	5.26 ± 0.21 b	8.66 ± 1.64 b

Ra: arithmetic mean roughness. Rms: quadratic mean roughness. Different letters (a–c) in the same column for each plate indicate significant difference (*p* < 0.05).

**Table 6:** Shannon and Equitability indexes of the sequence genera in biofilms of type A and Type B deposits on SSP.

Index	D1A	D3A	D5A	D1B	D3B	D5B
Shannon–Wiener (H′)	1.72	0.72	0.69	1.67	1.30	1.31
Equitability (J′)	0.52	0.21	0.21	0.50	0.39	0.39

D1A, D3A, D5A = Biofilms on type A deposits at 1, 3, and 5 days of maturation. D1B, D3B, D5B = Biofilms on type B deposits at 1, 3, and 5 days of maturation.

**Table 7:** Unifactorial design of AEW as cleaning agent, for 10 min at 30 °C.

Treatment	AEW (mg NaOH/L)	Reduction (log CFU/cm^2^)
1	50	5.97 ± 0.03 a
2	100	5.97 ± 0.04 a
3	200	5.97 ± 0.01 a
4	control	3.52 ± 0.10 b

Different letters in the column indicate significant difference (*p* < 0.05).

**Table 8:** Unifactorial design in the disinfection process with NEW at 20 °C for 5 min.

Treatment	Concentration of NEW (mg TAC/L)	Reduction (log CFU/cm^2^)
1	50	4.53 ± 0.24 a
2	100	4.30 ± 0.41 a
3	200	4.67 ± 0.01 a
4	control	2.63 ± 0.33 b

Different letters (a–b) in the column indicate significant difference (*p* < 0.05).

## Data Availability

Molecular data available on request.

## Supplementary Materials

The following are available online at https://www.mdpi.com/2304-8158/10/1/103/s1, Table S1: Complete factorial design 23 with 4 center points for the cleaning of SSP using AEW, Table S2: Microbial populations from each treatment of factorial design 23 with 4 center points, Table S3: Interactions of the factorial design 23.
