# Supplementary material for: Innovative Control of Biofilms on Stainless Steel Surfaces Using Electrolyzed Water in the Dairy Industry
Source: Foods. 2021 Jan 6;10(1):103. doi: 10.3390/foods10010103 (PMC7825347; doi:10.3390/foods10010103)
Supplement: Supplementary file 1 [file foods-10-00103-s001.pdf]

SUPPLEMENTARY MATERIAL

Table S1. Complete factorial design 2^3 with 4 center points for the cleaning of SSP using AEW

	Levels		
**Factor**	**-1**	**0**	**1**
Time (min)	10	20	30
AEW concentration (mg/L NaOH)	300	400	500
Temperature (°C)	30	40	50

The experimental unit consisted of SSP plates with type A deposits, and developed biofilms, because they showed microbial population high enough to evaluate the cleaning and disinfection process. All treatments received a final disinfection process using 200 ppm of NEW, at 20 °C for 5 min. For all treatments the response variable was microbial population by plate count agar for 24 h at 30°C.

Table S2. Microbial populations from each treatment of factorial design 2^3 with 4 center points.

AEW Concentration mg/L	Time min	Temperature °C	Log reduction/ cm^2
300	10	30	7.30
300	10	30	7.07
300	10	30	7.25
300	10	50	7.30
300	10	50	7.47
300	10	50	7.47
300	30	30	7.38
300	30	30	7.30
300	30	30	7.47
300	30	50	7.30
300	30	50	7.47
300	30	50	7.30
500	10	30	7.31
500	10	30	7.31
500	10	30	7.07
500	10	50	7.07
500	10	50	7.31
500	10	50	7.31
500	30	30	7.22
500	30	30	7.00
500	30	30	7.47
500	30	50	7.51
500	30	50	7.07
500	30	50	7.07

400	20	40	7.30
400	20	40	7.30
400	20	40	7.30
400	20	40	7.30
400	20	40	7.30
400	20	40	7.30
400	20	40	7.51
400	20	40	7.07
400	20	40	7.07
400	20	40	7.07
400	20	40	7.07
400	20	40	7.47

From statistical analysis, the experimental data were not fitted by the experimental model (p = 0.446), with $R^2 = 0.002$, while the lack of fit was significant (results not shown). Concentration of AEW was the factor with greater influence on microbial population, showing p=0.1086 (Table S3).

Table S3. Interactions of the factorial design 2^3

	Estimated value	Standard deviation	t Ratio	Prob>|t|
Intercept	7.281	0.032	226.72	<.0001*
AEW concentration	-0.055	0.032	-1.70	0.1086
Time	0.015	0.032	0.48	0.6377
Temperature	0.023	0.032	0.71	0.4858
Concentration*Time	-0.019	0.032	-0.58	0.5675
Concentration*Temperature	-0.026	0.032	-0.82	0.4257
Time*Temperature	-0.033	0.032	-1.02	0.3206
Concentration*Time*Temperature	0.029	0.032	0.92	0.3707
